# Comparison of Freestyle Optium Neo H and Centrivet GK Device in the Diagnosis of Hypoglycaemia and Hyperketonaemia in Dairy Goats: A Field Study

**DOI:** 10.1002/vms3.70159

**Published:** 2025-01-10

**Authors:** Murat Uztimür, Cennet Nur Ünal, Abdülkerim Deniz, Aytaç Pekmezci

**Affiliations:** ^1^ Faculty of Veterinary Medicine Department of Internal Medicine Bingöl University Bingol Turkey; ^2^ Free Researcher for Clinical Biochemistry İstanbul Turkey; ^3^ Department of Statistics Faculty of Science University of Muğla Sıtkı Koçman Muğla Turkey

**Keywords:** BHBA, diagnosis, goat, POC devices, pregnancy toxaemia

## Abstract

**Background:**

There is a lack of data on the validation and diagnostic performance of the Freestyle Optium Neo‐H (Freestyle) and Centrivet GK (Centrivet) devices for the diagnosis of hypoglycaemia, hyperglycaemia and hyperketonaemia in goats.

**Objectives:**

The aim of the present study was to validate the Freestyle and Centrivet for the analysis of whole blood beta‐hydroxybutyric acid (BHBA) and to validate the Freestyle for the analysis of whole blood glucose concentrations using the reference method (RM) in goat blood collected from the jugular and ear veins.

**Methods:**

Venous blood samples were utilised to assess glucose and BHBA concentrations using the Freestyle, Centrivet and RM. The cut‐off point of BHBA was ≥ 0.8 mmol/L for hyperketonaemia. A total of 198 paired blood samples (vena jugularis and ear vein) were collected from 99 hair goats. The cut‐off point for hypoglycaemia diagnosis was < 49 mg/dL.

**Results:**

There were proportional but no constant errors between RM and Freestyle and Centrivet for BHBA, and both proportional and constant errors were observed for glucose analysis. The mean bias for BHBA analysis was 0.14 and 0.06 mmol/L (Freestyle‐RM) 0.51 and 0.16 mmol/L (Centrivet‐RM) for jugular and ear veins, respectively. The mean bias for blood glucose analysis was 0.0 and 5.6 mg/L between Freestyle and RM in the jugular and ear veins, respectively. The sensitivity (Centrivet: 50%–61.3%; Freestyle: 93.6%–75.8%) and specificity (Centrivet GK: 75.7%–73%; Freestyle: 37.8%–70.3%) were determined in jugular and ear vein blood for hyperketonaemia diagnostics, respectively. The AUC of Freestyle was 0.89 and 0.95 in the jugular and ear vein for hypoglycaemia, respectively. The sensitivity of Freestyle was 60.3% and 96.8% in the jugular and ear vein for hypoglycaemia. The specificity of Freestyle was 100.0% and 76.7% for hypoglycaemia in jugular and ear veins, respectively.

**Conclusions:**

Freestyle demonstrated acceptable diagnostic performance for hypoglycaemia in ear veins, but neither Freestyle nor Centrivet showed sufficient diagnostic performance for hyperketonaemia. Both analysers were not interchangeable with RM in BHBA and glucose analysis.

## Graphical abstract

In this study, the diagnostic performance of Freestyle Optium Neo‐H and Centrivet GK devices in hyperketonaemia and hypoglycaemia or hyperglycaemia goats was evaluated. Freestyle demonstrated acceptable diagnostic performance for hypoglycaemia in ear veins, but neither Freestyle nor Centrivet showed sufficient diagnostic performance for hyperketonaemia. The findings suggest a need for future studies focused on the development of Freestyle Optium Neo‐H and Centrivet GK chips and test kits specifically tailored for goats.

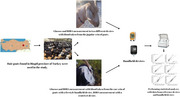

## 1 Introduction

The periparturient period in small ruminants represents a critical phase for both maternal and neonatal health. The disturbances in carbohydrate and lipid metabolism were frequently documented during this period, particularly within the final six weeks of gestation (Rook 2000; Brozos, Mavrogianni, and Fthenakis 2011). These metabolic disorders lead to a negative energy balance (NEB) in the animal, resulting in pregnancy toxaemia. The incidence of pregnancy toxaemia is reported to range from 35% to 88.9%, with mortality rates escalating to as high as 80% in the absence of therapeutic intervention (Brozos, Mavrogianni, and Fthenakis 2011; Rook 2000). Furthermore, it is noteworthy that to date no definitive cut‐off point for BHBA concentration has been established to indicate hyperketonaemia (HK) in dairy goats.

In small ruminants, blood concentrations of BHBA below 0.8 mmol/L are considered normal, whereas levels at or above this threshold (BHBA ≥ 0.8 mmol/L) suggest an NEB due to compromised energy metabolism (Bani Ismail et al. 2008; Pichler et al. 2014a; Rook 2000; Schwendenwein 2009). The semi‐quantitative dipsticks for the detection of urinary or milk ketone bodies in small ruminants are available; however, their sensitivity was limited (Brozos, Mavrogianni, and Fthenakis 2011). The wet‐biochemistry method is the preferred reference method (RM) for quantifying BHBA concentrations in cases of pregnancy toxaemia or ketosis (Panousis et al. 2018; Pichler, Damberger, Schwendenwein, et al. 2014b; Uztimür, Gazioğlu, and Yilmaz 2024).

Recently, point‐of‐care (POC) devices have gained prominence in the veterinary care of both large and small ruminants (Panousis et al. 2018; Uztimür and Ünal 2024). These devices are increasingly favoured for measuring blood glucose and BHBA concentrations due to their compact design, cost‐effectiveness, ease of use and rapid output. Their utility is significant in the veterinary clinics and on farms, particularly for the diagnosis of conditions such as hypoglycaemia or HK. Numerous validated POC devices are available for the diagnosis of ketosis or hypoglycaemia in ruminants (Aksoy et al. 2023; Lopes, Valldecabres, and Silva‐del‐Río 2019; Panousis et al. 2018), but their validation in goats is less common (Panousis et al. 2018; Pichler, Damberger, Schwendenwein, et al. 2014b). The Freestyle Optium Neo‐H is tailored for human‐specific measurements of blood glucose and BHBA concentrations, whereas the Centrivet GK is bovine‐specific for these parameters. No studies have yet been conducted with the Freestyle Optium Neo‐H and Centrivet GK devices for the analysis of blood BHBA and glucose concentrations in dairy goats.

The objective of the present study is to validate the Freestyle Optium Neo‐H and Centrivet GK POC devices for HK diagnosis by the analysis of BHBA concentration in caprine blood drawn from the jugular and ear veins and to compare the Freestyle Optium Neo‐H with the RM for the analysis of blood glucose concentration.

## 2 Materials and Methods

### 2.1 Animals

This investigation was carried out on a cohort of 99 goats of varied breeds, located across 10 different farms within the Bingöl Province in Türkiye. A total of 99 samples were used for this study. The number of samples was sufficient in studies comparing two different methods (Jensen and Kjelgaard‐Hansen 2006; Bilic‐Zulle 2011). The goats in the study were in the last trimester of pregnancy, consisting of hair goats (*n* = 69), Aleppo goats (*n* = 25) and Saanen goats (*n* = 5), aged between 1.5 and 6 years, and having an average lactation number of 4.3. The weather condition during the study was 4°C to 15°C. Blood collection and analysis with POC devices were performed by the same person according to the instructions of the POC device manufacturers.

### 2.2 Analysis of Blood Glucose and BHBA Concentrations

A single blood collection was executed from the jugular vein (vena jugularis) and ear vein of the goats. The Freestyle Optium Neo‐H device, developed for human use and provided by Abbott Diabetes Care, Witney, UK, was used in conjunction with the bovine‐specific Centrivet GK device from ACON Laboratories, Inc. of San Diego, USA. BHBA concentration in blood was measured using one drop of whole blood obtained by minimally invasive venipuncture from the jugular and ear veins, applied to disposable test strips compatible with both the Centrivet GK and Freestyle Optium Neo‐H devices. In this article, it was evaluated whether there was a difference between the measurement results of the ear vein compared to the jugular vein, and its potential to be used in the measurement of glucose and BHBA was investigated. Measurements were performed after the calibration of both BHBA and glucose analysis on the Freestyle Optium Neo‐H and BHBA on the Centrivet GK devices. According to the manufacturer's specifications, the Freestyle Optium Neo‐H device provides glucose readings between 20 and 500 mg/dL in 5 s and BHBA readings from 0.0 to 8.0 mmol/L in 10 s, with a ‘LO’ warning for glucose concentrations below these ranges. The Centrivet device, when applied with 1.2 µL of blood, yields BHBA results between 0.0 and 8.0 mmol/L in 10 s. All glucose and BHBA measurements were consistently conducted by the same person. During glucose measurements with the Freestyle Optium Neo‐H device, a ‘LO’ message was observed in one sample from the jugular vein and in six samples from the ear vein.

For the analysis of serum BHBA concentration, 5 mL of blood samples were collected from vena jugularis using anticoagulant‐free tubes (BD Vacutainer, Plymouth, UK), while 3 mL of blood samples were obtained using sodium fluoride potassium oxalate‐containing tubes (Vacusel, Turkey) for the analysis of glucose concentration. After these samples were delivered to the laboratory, they were kept at an ambient temperature of approximately 20°C for approximately 1 h and then centrifuged (Hermle Z 366, Germany) at 2200 × *g* for 10 min at room temperature to separate serum and plasma. These serum and plasma samples were then stored at −20°C until analysis. The analysis of serum BHBA and plasma glucose concentrations was conducted within two months after the completion of blood collection. An automated biochemistry analyser (RX Monaco, Randox Laboratories) was used for the determination of serum BHBA concentration, employing the Randox D‐3 Hydroxybutyrate Ranbut reagent, and this method was the RM for BHBA analysis. This method principally involves the oxidation of d‐3‐hydroxybutyrate to acetoacetate through the action of the enzyme 3‐hydroxybutyrate‐dehydrogenase. This oxidation process is linked to a NAD indicator system, and the resulting colour change is proportional to the BHBA concentration.

The analysis of plasma glucose concentration was performed using an automated biochemistry device (Mindray BS‐2000m, China), employing the photometric principle based on the hexokinase enzyme reaction as the RM. In this process, hexokinase catalyses the phosphorylation of glucose by ATP into glucose 6‐phosphate and adenosine diphosphate, accompanied by a colorimetric change in NADP. To determine the intra‐analytical coefficient of variation (CV), the low, medium and high concentrations were measured eight consecutive times for both the Centrivet GK and Freestyle Optium Neo‐H instruments and 10 times for the RMs (Pichler, Damberger, Arnholdt, et al. 2014a). The CV was calculated using the formula: CV = (standard deviation/mean) × 100.

### 2.3 Statistical Analysis

MedCalc software (MedCalc Software Ltd, Belgium) version 2022 was used to perform the statistical analyses. Normality of the data was evaluated using the Shapiro−Wilk test. Descriptive statistical analyses were performed for median, minimum and maximum concentrations of blood BHBA and glucose concentrations and for the prevalence of HK, hypoglycaemia and hyperglycaemia for all devices. The agreement between the results of the RM and the POC devices was evaluated through a Bland–Altman plot of the agreement method (Bland and Altman 1986). To ascertain constant bias and linearity between the POC devices and the RMs, Passing−Bablok regression equation analysis was used to compare the test sets (Jensen and Kjelgaard‐Hansen 2006; Bilic‐Zulle 2011). The adjusted Passing−Bablok regression obscures the 45‐degree line (Y = X) in the case of perfect agreement between the methods. HK was delineated as a BHBA concentration of ≥ 0.8 mmol/L based on the results of the RM (Panousis et al. 2018; Pichler, Damberger, Schwendenwein, et al. 2014b). Receiver operating characteristic (ROC) analysis was conducted to diagnose HK, hypoglycaemia and hyperglycaemia by Freestyle Optium Neo‐H and Centrivet GK based on the results of the RM. Samples were categorised as hypoglycaemic (cut‐off point of blood glucose < 49 mg/dL) and hyperglycaemic (cut‐off point of blood glucose > 63 mg/dL) in ROC analysis based on the results from the RM (Pichler, Damberger, Schwendenwein, et al. 2014b; Schwendenwein 2009). Utilising this classification and established cut‐off points, the sensitivity, specificity and area under the curve (AUC) were calculated in the ROC analysis. The Cohen's kappa coefficient was calculated for the inter‐rater reliability of variables (≤ 0.00 no agreement, 0.01–0.20 nor or slight, 0.21–0.40 fair, 0.41–0.60 moderate, 0.61–0.80 substantial, 0.81–1.00 perfect agreement) to observe the agreement rates (Cohen 1960). The number of samples at each defined cut‐off point was presented as an absolute number (*n*) to show the distribution frequency of samples for serum BHBA and plasma glucose concentration analysed by RM.

## 3 Results

A total of 198 paired blood samples (from both the vena jugularis and ear vein) were collected from 99 hair goats. Six samples (five from vena jugularis and one from the ear vein) were excluded from analysis of plasma glucose concentration from the statistical evaluation due to the ‘LO’ warning displayed when measured with the Freestyle Optium Neo‐H. Consequently, blood glucose levels were analysed in 93 goats only.

### 3.1 Intra‐Assay CV

**FIGURE 1:**
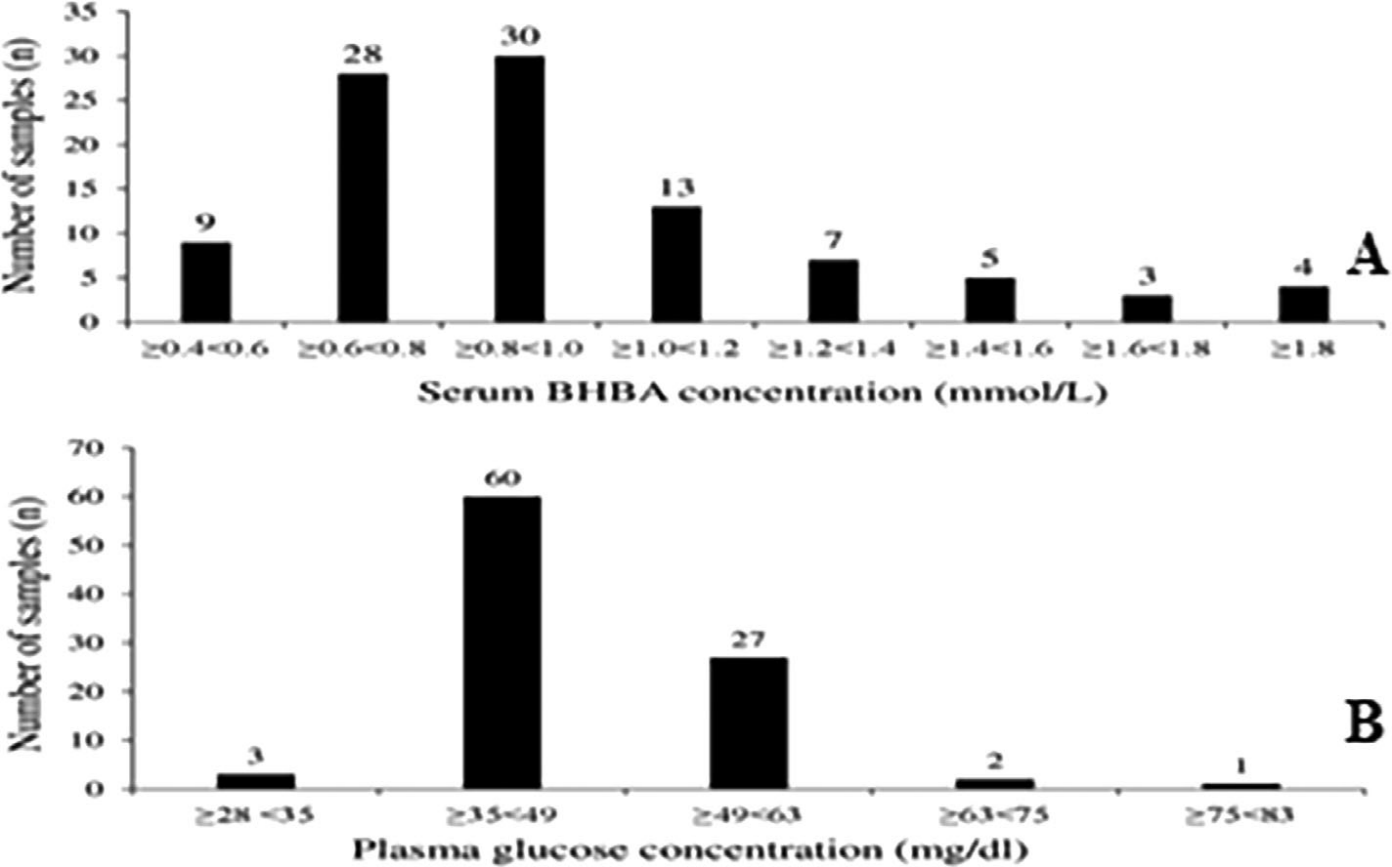
Number of samples at different cut‐off points of serum beta‐hydroxybutyric acid (A) (BHBA) (*n* = 99) and plasma glucose concentrations (B) (*n* = 93) analysed by reference method in dairy goats.

CVs were tested for low, medium and high concentrations of BHBA in serum (RM) and whole blood (Freestyle Optium Neo‐H and Centrivet GK). CV in BHBA analysis was 10.71%, 3.70% and 13.96% for Freestyle Optium Neo‐H; 11.11%, 7.75% and 3.71% for Centrivet GK; and 2.38%, 1.20% and 0.31% for RM. CVs were tested for low and high blood glucose concentrations in plasma and whole blood. CV in glucose analysis was 4.88% and 9.56% for Freestyle Optium Neo‐H and 0.39% and 1.88% for RM. Distribution plots of samples analysed by RM, serum BHBA and plasma glucose concentration are presented in Figure 1.

### 3.2 Analysis of BHBA Concentration

The median, minimum and maximum serum concentrations of BHBA across all samples, as measured by the RM and POC devices, were presented as descriptive statistics in Table 1. In addition, 37.4% (*n* = 37) of the samples were determined to be less than 0.8 mmol/L, while 62.2% (*n* = 62) were equal to or greater than 0.8 mmol/L (Figure 1).

**TABLE 1:** Median, minimum and maximum values of serum BHBA (*n* = 99) and plasma glucose concentrations (*n* = 93) analysed with the reference method and whole blood BHBA and glucose concentrations analysed with the test devices Freestyle Optium Neo‐H and Centrivet GK in dairy goats.

Device	Anatomical location	BHBA concentration (mmol/L)	Glucose concentration (mg/dL)
Reference method	VJ^a^	0.87 (0.41–3.17)	45 (28.1–82.8)
Freestyle Optium Neo‐H	VJ^b^	0.90 (0.7–3.2)	43 (20–165)
	EVB	0.90 (0.4–2.6)	39 (23–87)
Centrivet GK	VJ^b^	1.4 (0.8–3.7)	—
	EVB	1 (0.3–3.9)	—

Abbreviation: EVB, ear venous blood (for BHBA and glucose analysis in whole blood).

^a^ VJ: Blood taken from vena jugularis (serum for BHBA, plasma for glucose analysis).

^b^ VJ: A drop of whole blood from vena jugularis for BHBA analysis.

**FIGURE 2:**
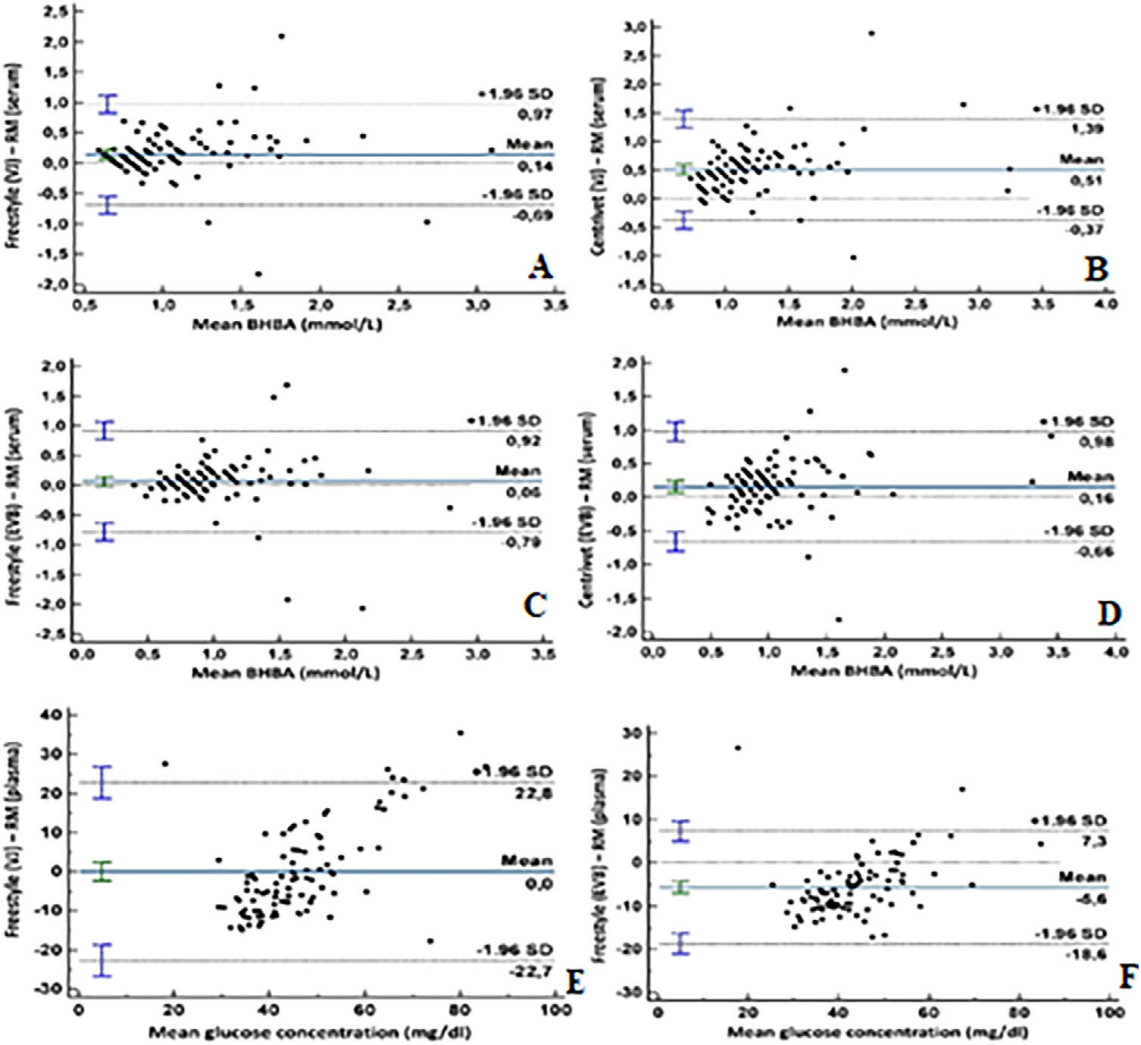
Bland−Altman plots of agreement for blood beta‐hydroxybutyric acid (BHBA) and glucose concentrations between reference method (RM) by wet‐biochemistry using Randox kits and Freestyle Optium Neo‐H (Freestyle) and Centrivet GK (Centrivet) in the samples from vena jugularis (VJ) and ear vein blood (EVB) in dairy goats. (A) BHBA; Freestyle (VJ)‐RM (Serum), (B) BHBA; Centrivet (VJ)‐RM (Serum), (C) BHBA; Freestyle (EVB)‐RM (Serum), (D) BHBA; Centrivet (EVB)‐RM (Serum), (E) Glucose; Freestyle (VJ)‐RM (Plasma), (F) Glucose; Freestyle (EVB)‐RM (Plasma).

**FIGURE 3:**
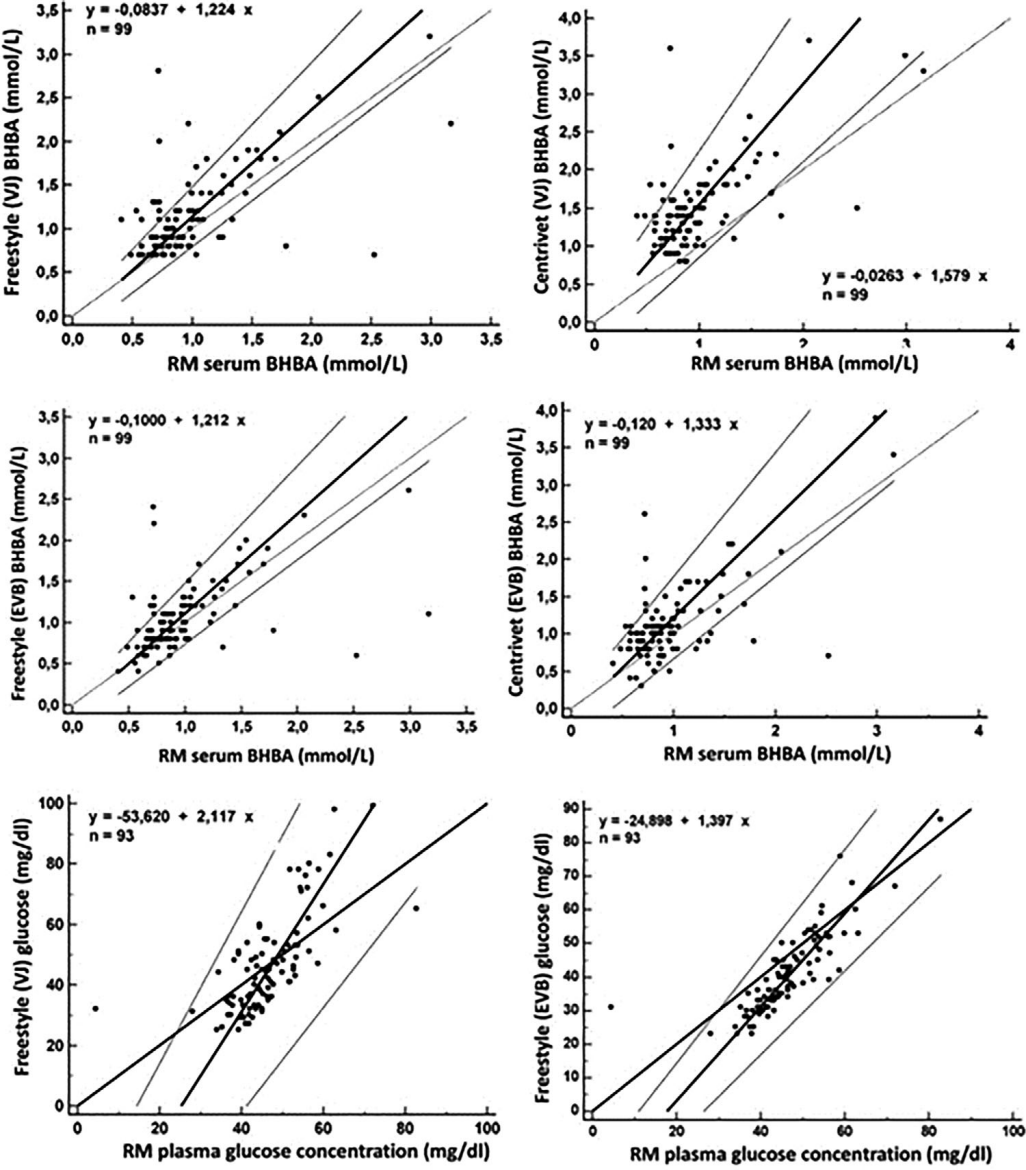
Passing−Bablok regression equations for serum beta‐hydroxybutyric acid (BHBA) and plasma glucose concentrations between reference method (RM) by wet‐biochemistry using Randox kits and Freestyle Optium Neo‐H (Freestyle) and Centrivet GK (Centrivet) in the samples from vena jugularis (VJ) and ear vein blood (EVB) in dairy goats.

**FIGURE 4:**
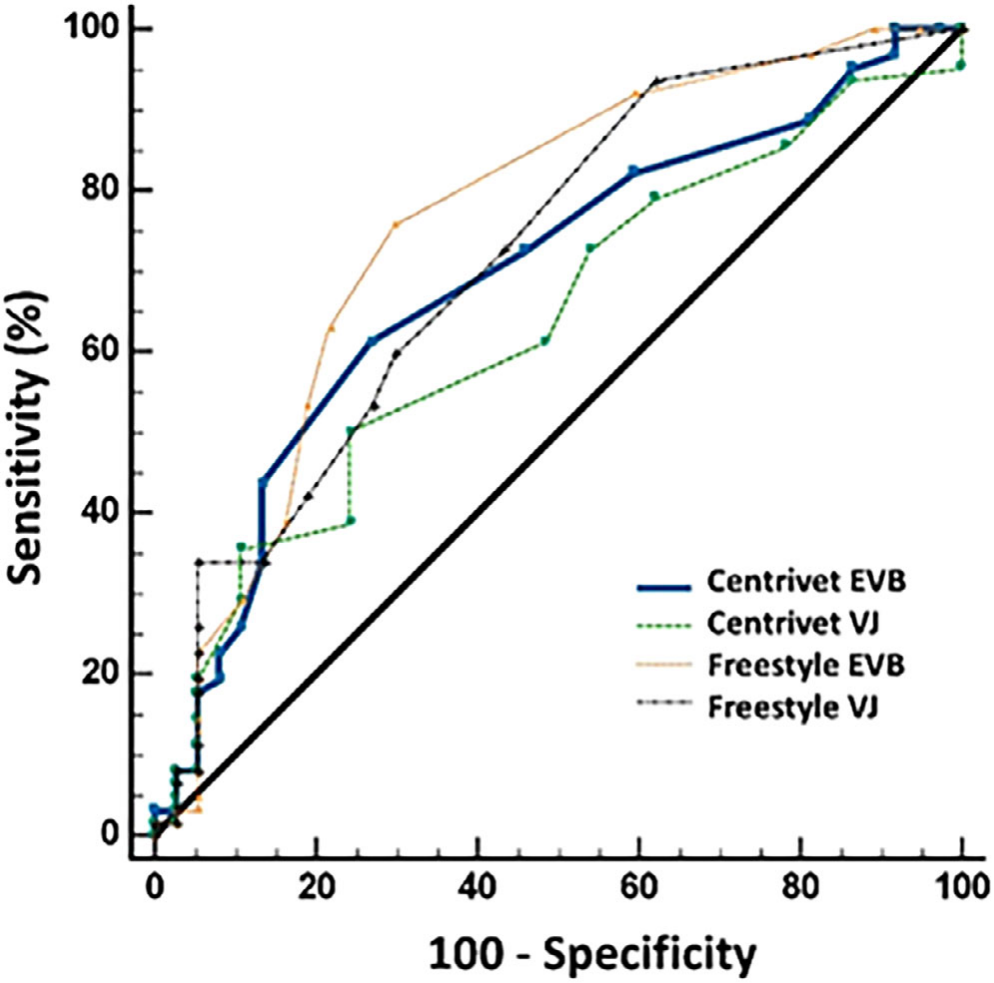
Receiver operating characteristic curves for hyperketonaemia diagnosis (serum beta‐hydroxybutyric acid concentration ≥ 0.8 mmol/L by reference method wet biochemistry using Randox kits) by Freestyle Optium Neo‐H (Freestyle) and Centrivet GK (Centrivet) in the samples from vena jugularis (VJ) and ear vein blood (EVB) in dairy goats.

Freestyle Optiumun, in terms of the Cohen's kappa coefficient, the comparison between the RM and the Freestyle yielded values of 0.12 for vena jugularis and 0.44 for ear vein samples. Cohen's kappa coefficients were 0.34 for vena jugularis and 0.26 for ear vein samples between the RM and the Centrivet device. Figure 2 presented the Bland–Altman plots of agreement for both the Freestyle Optium Neo‐H and Centrivet GK devices in comparison to the RM. These plots provided a visual representation of the mean and total errors, along with the 95% confidence intervals (CIs) for blood samples obtained from both the ear vein and jugular vein. Figure 3 presented the Passing−Bablok regression equation for the analysis of BHBA with the Freestyle Optium Neo‐H and Centrivet GK devices in comparison to the RM. For the Freestyle Optium Neo‐H device, the intercept was determined between 95% CI: −0.25 to 0.05 for vena jugularis and −0.28 to 0.05 for ear vein, while the slope value was determined between 95% CI: 1.05–1.42 for vena jugularis and 95% CI: 1.02–1.42 for ear vein. There were no constant errors, but there were proportional errors between the RM and Freestyle Optium Neo‐H in both jugular and ear vein samples. For the Centrivet GK device, the intercept was determined between 95% CI: −0.40 to 0.25 for vena jugularis and 95% CI: −0.43 to 0.10 for ear vein, while the slope value was between 95% CI: 1.25–2.00 for vena jugularis and 95% CI: 1.09–1.66 for ear vein. There were proportional errors but no constant errors between the RM and Centrivet GK in both jugular and ear vein samples. The ROC analysis, as shown in Table 2 and Figure 4, revealed that the sensitivity, specificity and AUC values for the Freestyle Optium Neo‐H and Centrivet GK devices in detecting HK were considerably low for both jugular and ear vein samples.

**TABLE 2:** Receiver operating characteristic results for hyperketonaemia and hypoglycaemia based on the cut‐off points of reference method.

Parameter	Device	Anatomic location	AUC (95% CI)	Sensitivity % (95% CI)	Specificity % (95% CI)	AC
Hyperketonaemia (serum BHBA ≥ 0.8 mmol/L)	Freestyle	Vena jugularis	0.717 (0.618–0.803)	93.6 (84.3–98.2)	37.8 (22.5–55.2)	> 0.7
	Freestyle	Ear vein	0.759 (0.663–0.839)	75.8 (63.3–85.8)	70.3 (53.0–84.1)	> 0.8
	Centrivet	Vena jugularis	0.632 (0.529–0.727)	50.0 (37.0–63.0)	75.7 (58.8–88.2)	> 1.4
	Centrivet	Ear vein	0.692 (0.591–0.781)	61.3 (48.1–73.4)	73.0 (55.9–86.2)	> 1.0
Hypoglycaemia (plasma glucose < 49 mg/dL)	Freestyle	Vena jugularis	0.893 (0.811–0.957)	60.3 (49.0–79.0)	100.0 (70.8–100.0)	≤ 40.0
	Freestyle	Ear vein	0.948 (0.881–0.983)	96.8 (79.0–99.0)	76.7 (60.8–88.9)	≤ 50.3

Abbreviations: AC, associated criterion; AUC, area under the curve; BHBA, beta‐hydroxybutyric acid; Centrivet: Centrivet GK; Freestyle, Freestyle Optium Neo‐H.

### 3.3 Analysis of Blood Glucose Concentration

Descriptive statistics of plasma glucose concentration measured by the RM and whole blood glucose concentration analysed by Freestyle Optium Neo‐H were presented in Table 1. 69.69% of samples were hypoglycaemic, 27.27% normoglycaemic and 3.03% hyperglycaemic (Figure 1). According to Bland–Altman plots of agreement (Figure 2), the mean and total error for the Freestyle Optium Neo‐H in vena jugularis blood samples were 0.00 and 45.5 mg/dL, respectively, while for ear vein samples, the mean and total error were −5.6 and 25.9 mg/dL. Passing−Bablok regression analysis (Figure 3) revealed constant and proportional errors. The intercept 95% CI was −72.10 to −36.48 for the jugular vein; it was −33.17 to −17.62 for the ear vein. The slope 95% CI was 1.74–2.51 for the jugular vein and 1.25–1.59 for the ear vein. Neither the intercept did not include the value zero nor the slope did not include the value 1.0.

**FIGURE 5:**
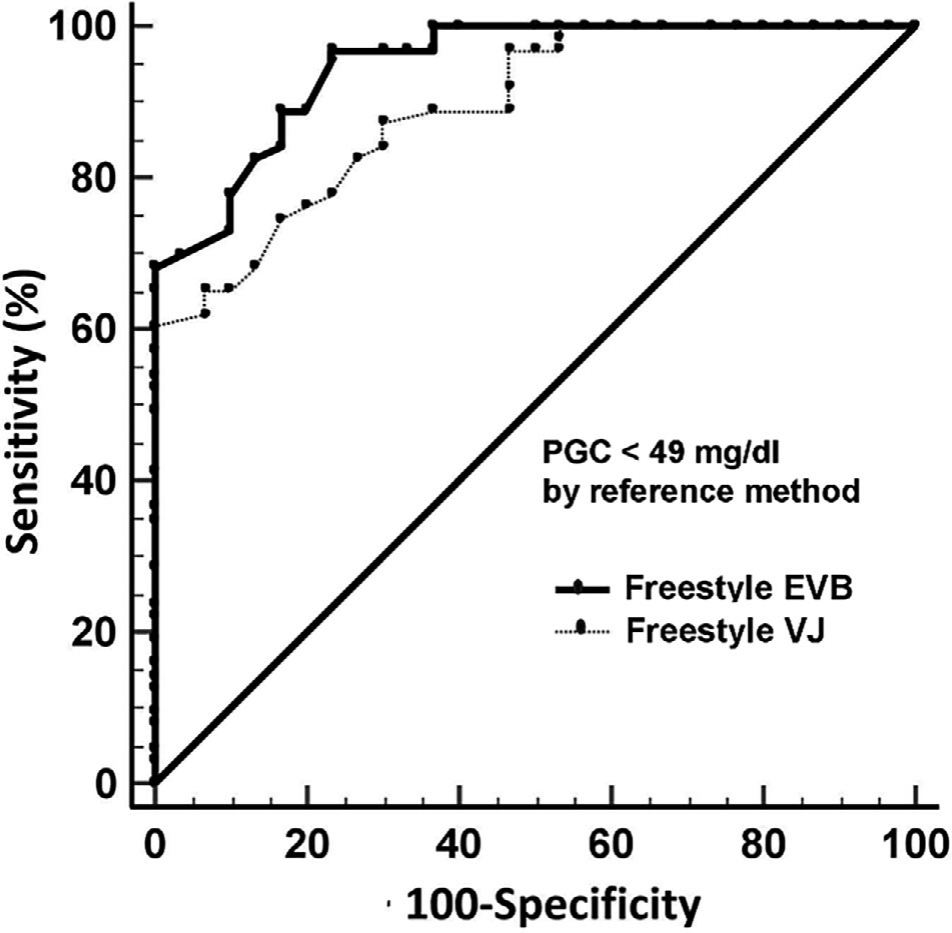
Receiver operating characteristic curves for hypoglycaemia diagnosis (plasma glucose concentrations (PGC) < 49 mg/dl by reference method: wet biochemistry) for Freestyle Optium Neo‐H (Freestyle) in the samples from vena jugularis (VJ) and ear vein blood (EVB) in dairy goats.

Results from ROC analysis (Table 2; Figure 5) indicated that AUC values were above 0.893 and 0.948 for detecting hypoglycaemia in both ear and jugular vein samples, demonstrating good diagnostic accuracy of Freestyle Optium Neo‐H. In identifying hypoglycaemia, the sensitivity of the Freestyle Optium Neo‐H was much better in ear vein samples than jugular vein samples. A ROC analysis was not performed due to the low number of hyperglycaemic cases (*n* = 3).

In terms of the Cohen's kappa coefficient, the comparison between the RM and the Freestyle yielded below 0.10, which indicated poor agreement in glucose analysis.

## 4 Discussion

POC devices gained substantial usage in veterinary medicine in recent years, primarily due to their ability to deliver rapid results on the farm, along with their cost‐effectiveness and portability. The majority of validation studies for these devices have predominantly focused on cattle, with only a limited number addressing their accuracy in sheep and goats (Lopes, Valldecabres, and Silva‐del‐Río 2019; Panousis et al. 2018; Pichler, Damberger, Schwendenwein, et al. 2014; Tümer and Kılınç 2023; Jones et al. 2018). The aim of the current study was therefore to validate and evaluate the diagnostic accuracy of the Freestyle Optium Neo‐H and Centrivet GK devices for the determination of BHBA concentration in dairy goats in the last 3 weeks of pregnancy and for the comparative analysis of blood glucose concentration with Freestyle Optium Neo‐H. Notably, there has been no prior validation of blood BHBA and glucose measurements using the Freestyle Optium Neo‐H, nor BHBA measurements with Centrivet GK in dairy goats. While the Centrivet GK device used in the present research is marketed for use in cattle, its validation and diagnostic accuracy in goats were also examined for the analysis of whole blood BHBA concentration. It is important to note that Freestyle Optium Neo‐H kits are originally designed for human use.

The core objective of method comparison studies is to scrutinise the efficacy of novel analytical approaches in contrast to established, widely utilised methodologies and to determine their congruence with standard RMs (Lopes, Valldecabres, and Silva‐del‐Río 2019; Bilic‐Zulle 2011). Such studies predominantly incorporate Bland–Altman and Passing−Bablok regression equation analysis. The latter, a non‐parametric statistical test, is employed to evaluate potential systemic discrepancies between the data sets and the alignment of the analytical approach (Bilic‐Zulle 2011). As delineated by Passing and Bablok (1983), an intercept encompassing zero and a slope containing 1.0 within a 95% CI denote methodological equivalence, suggesting the interchangeability of the two methods (Passing and Bablok 1983; Bilic‐Zulle 2011). The present research marks a novel application of the Passing−Bablok regression analysis for assessing BHBA measurements in dairy goats, utilising POC devices on jugular and ear vein whole blood samples compared to the RM in serum samples. Previous investigations into BHBA analysis performance of various ketometers in dairy cows indicated that Precision Xtra exhibited negative constant and positive proportional biases in the Passing−Bablok regression analysis (Yepes et al. 2018). Similarly, Freestyle demonstrated positive constant and proportional biases in sheep (Tümer and Kılınç 2023), while the Freestyle Precision Neo ketometer did not exhibit negative constant and positive proportional biases (Jansen et al. 2021). In alignment with these findings, the present study revealed that both the Freestyle Optium Neo‐H and Centrivet GK ketometers exhibited no constant errors but proportional errors in measurements of whole blood BHBA obtained from jugular and ear veins, suggesting non‐interchangeability with the RM.

Bland–Altman analysis, another pivotal technique in method comparison studies, has been used to assess the agreement between disparate methodologies and to appraise the deviation between mean differences (Bilic‐Zulle 2011). In research conducted by Jones et al. (2018) on late‐pregnant ewes, the Nova Vet meter was found to overestimate blood BHBA concentrations by 0.128 mmol/L. Another study on ewes at the culmination of pregnancy indicated average biases of 0.224 and 0.737 mmol/L for the Freestyle Optium Neo H and Taidoc‐TD4235 ketometers, respectively (Tümer and Kılınç 2023). Pichler, Damberger, Schwendenwein, et al. (2014b) reported mean biases of −0.12 and −0.21 mmol/L for ear and jugular blood BHBA concentrations in goats using the Freestyle Precision ketometer. In the present study, the mean bias of the Freestyle Optium Neo‐H device in ear and jugular vein samples was found to be consistent with those reported for the Freestyle Precision and Freestyle Optium Neo H (Tümer and Kılınç 2023) ketometers. The investigation found that the whole blood BHBA ear vein value measured by the Centrivet GK device closely aligned with the average bias of the FreeStyle Precision (Pichler, Damberger, Schwendenwein, et al. 2014b), Nova Vet ketometer (Jones et al. 2018) and WellionVet BELUA (Panousis et al. 2018) ketometers. Both the Freestyle Optium Neo‐H and Centrivet GK devices overestimated blood BHBA concentrations, with the bias amplifying as BHBA concentration increased. The calculated total biases of 95% CI of Freestyle Optium Neo‐H and Centrivet in both ear and jugular vein blood samples exceeded the accepted threshold of BHBA 0.80 mmol/L for HK. The kappa coefficient presented slight or moderate agreement between the RM and Freestyle Optium Neo‐H and Centrivet GK. According to McHugh (2012), the kappa coefficient indicated much less, if any, agreement at all.

Numerous investigations involving sheep (Pichler, Damberger, Schwendenwein, et al. 2014b) and goats (Doré et al. 2013; Pichler, Damberger, Arnholdt, et al. 2014a) have documented a robust correlation between various ketometer devices and the established gold standard method. In the present study, a moderate yet significant correlation was observed between the whole blood BHBA concentrations measured by the Freestyle Optium Neo‐H and Centrivet GK devices and the RM. This finding diverges somewhat from the strong correlations reported in the aforementioned studies. However, it is important to note that studies in dairy cows have highlighted instances where a high correlation does not necessarily imply compatibility between the RM and the test device (Deniz et al. 2023; Mert et al. 2023). This discrepancy can be attributed to the nature of linear correlation analyses, which, in contrast to Passing−Bablok regression, focus primarily on random deviations, so that a high significant correlation does not indicate good agreement (Giavarina 2015). In a study (Jones et al. 2018), the diagnostic accuracy, sensitivity and specificity values of the Nova Vet ketometer were found to be above 90% for detecting HK at a BHBA cut‐off point of ≥ 0.8 mmol/L in sheep. Meanwhile, in another study utilising the WellionVet BELUA ketometer in goats, sensitivity and specificity values were reported to range between 50% and 100% (Panousis et al. 2018). In the present research, the Freestyle Optium Neo‐H device, specifically when used to test ear vein samples, demonstrated the highest diagnostic accuracy for the detection of HK (sensitivity = 75.8%, specificity = 70.3%, AUC = 0.76, SUM of se + sp = 146.1%). All statistical analyses used in the present study indicated no interchangeability of Freestyle Optium Neo‐H and Centrivet GK with the RM in the measurement of blood BHBA in dairy goats. The low diagnostic performance of the devices in the diagnosis of HK in the study may be related to the fact that the optimal ambient temperature for both the Freestyle Optium Neo H (Temperature: +4, +30) and the Centrivet GK devices (Temperature: +5, +45) may affect the measurements. In addition, there may be effects such as the sample size of the study, seasonal conditions and operator differences in sample collection and technique, as well as the batch number of the strips.

In line with the ‘bioanalytical method validation guide’ published by the European Medicines Agency (2011), low, medium and high BHBA concentrations were selected to determine CV in the present study. In the study conducted by Tümer and Kılınç (2023), it was reported that CVs in the low, medium and high BHBA concentrations for the Freestyle Optium Neo H device were 14.28%, 6.25% and 8.97%, respectively. The same researchers reported that the low, medium and high CVs of the Taidoc device were 14.86%, 14.95% and 18.10%, respectively (Tümer and Kılınç 2023). In this study, it was determined that the low and middle values of the Freestyle Optium Neo H device were lower than the CV values found in both devices by Tümer and Kılınç (2023). In addition, in this study, eight consecutive measurements were performed to determine CV, and more measurements were performed than Lopes, Valldecabres, and Silva‐del‐Río (2019) and Tümer and Kılınç (2023). In the present study, CV values > 10% are thought to be related to the devices, test strips and the environment in which the analysis was performed.

Hypoglycaemia and hyperglycaemia are prevalent conditions in goats during the final weeks of pregnancy, and their early and rapid diagnosis is crucial for maintaining the health of goats (Souto et al. 2013; Souza et al. 2020; Iqbal et al. 2022). According to established thresholds, a blood glucose concentration greater than 63 mg/dL is indicative of hyperglycaemia, while levels below 49 mg/dL are classified as hypoglycaemic in goats (Schwendenwein 2009; Pichler, Damberger, Arnholdt, et al. 2014a; Pichler, Damberger, Schwendenwein, et al. 2014b). In a study evaluating the efficacy of the Precision Xceed device in sheep, it was found to have very low diagnostic performance for detecting hypoglycaemia and hyperglycaemia (Pichler, Damberger, Schwendenwein, et al. 2014b). Conversely, a study utilising the Freestyle Precision device in goats reported that measurements from the ear vein and vena jugularis were significantly more effective in detecting hyperglycaemia than hypoglycaemia (Pichler, Damberger, Arnholdt, et al. 2014a). Echoing the findings of Pichler, Damberger, Schwendenwein, et al. (2014b), the present study did not perform ROC analysis due to the low number of hyperglycaemic samples. Although the diagnostic accuracy of Freestyle Optium Neo‐H was very good to excellent in jugular and ear veins, the sensitivity was very low in jugular veins compared to the high sensitivity in ear vein samples. The possible reason why the sensitivity of Freestyle Optium Neo‐H is much better in ear vein samples than in jugular vein samples may be related to the greater number of hypoglycaemic samples in the ear vein. The Freestyle Optium Neo‐H device, requiring only a single drop of blood, proved effective in acceptably identifying both hypoglycaemia across these anatomical locations.

A study focusing on hyperketonaemic sheep by employing the Bland–Altman method found that the average bias for glucose measurement was 8.4 mg/dL in vena jugularis and 17.1 mg/dL in the ear vein, indicating an overestimation of values (Pichler, Damberger, Schwendenwein, et al. 2014b). Similarly, a study in dairy goats using the Freestyle Precision glucometer reported a mean bias of −6.3 mg/dL in the ear vein and −10.9 mg/dL in the vena jugularis, thereby overestimation the RM (Pichler, Damberger, Arnholdt, et al. 2014a). In line with these previous findings, the present study observed a mean positive bias of −5.6 mg/dL in the ear vein; however, in contrast, no mean bias (0.0 mg/dL) in the jugular vein was observed. The total bias was lower in blood samples taken from the ear vein compared to those from the jugular vein. Notably, while the mean bias was 0.0 in the jugular vein, the total bias exceeded 45 mg/dL, significantly surpassing the acceptable total error level of 5.6%–6.96% (Westgard 2015). Meaningfully, a low or zero mean bias did not necessarily indicate an agreement between two methods as shown in the present study.

No research to date has evaluated glucose concentrations in hyperketonaemic dairy goats using the Passing−Bablok regression method, making this study the inaugural one to assess blood glucose concentration in such a context using the Freestyle Optium Neo‐H device and the Passing−Bablok method. In a study by Megahed et al. (2015) utilising the Precision Xtra device, both proportional and constant errors were identified. Similarly, an investigation examining the agreement between six different glucometers and the RM in dairy cows found constant and proportional errors in the results (Lopes, Valldecabres, and Silva‐del‐Río 2019). In alignment with these findings, the present study identified a negative constant and positive proportional error in the Freestyle Optium Neo‐H blood glucose measurements. In addition, Cohen's kappa coefficients confirmed this disagreement with a value below 0.10. This outcome indicated that the Freestyle Optium Neo‐H was not interchangeable with the RM for the accurate determination of glucose concentration. A potential explanation for the deviation in glucometer readings observed in this study could be attributed to the fact that the POC device used, designed for human use, may not be entirely suitable for goats. This discrepancy could stem from the higher haematocrit value found in humans compared to goats, affecting the accuracy of glucometer readings in non‐human samples. However, any interaction of blood glucose with haematocrit is the objective of the further studies in goats.

### 4.1 Limitations and Conclusion

This study has certain limitations. First, the climatic conditions and the small number of hyperketonaemic and hyperglycaemic samples may be limiting factors in the current study. Second, as this is a field study, the likelihood of within‐group variation is greater, and multiple influencing factors on individual animals are also possible.

In conclusion, the present research demonstrated that the Freestyle Optium Neo‐H device exhibited good diagnostic accuracy in detecting hypoglycaemia in goats from ear vein samples, despite not being in complete agreement with the RM. However, both the Freestyle Optium Neo‐H and Centrivet GK devices showed suboptimal performance in terms of sensitivity and specificity for the detection of HK in goats. Moreover, they cannot be used interchangeably with the RM due to their lack of compatibility. The findings suggest a need for future studies focused on the development of Freestyle Optium Neo‐H and Centrivet GK chips and test kits specifically tailored for goats. Such advancements would facilitate more accurate measurements of glucose concentrations alongside BHBA, enhancing the diagnostic capabilities in veterinary medicine.

## Author Contributions

**Murat Uztimür**: conceptualisation, investigation, writing–original draft, methodology, writing–review and editing, resources, supervision. **Cennet Nur Ünal**: project administration, investigation, writing–review and editing. **Abdülkerim Deniz**: validation, supervision, writing–review and editing. **Aytaç Pekmezci**: validation, writing–review and editing.

## Ethics Statement

The research received approval from the Bingöl University Experimental Animals Ethics Committee (Date and Number: 31.03.2023 ‐02/04).

## Conflicts of Interest

The authors declare no conflicts of interest.

### Peer Review

The peer review history for this article is available at https://publons.com/publon/10.1002/vms3.70159.

## Data Availability

The data that support the findings of this study are available from the corresponding author upon reasonable request.
